# Analysis of the characteristics of blood inflammatory cytokines and their influencing factors in acute exacerbations of allergic asthma in children

**DOI:** 10.3389/fped.2025.1571556

**Published:** 2025-04-07

**Authors:** Ren Shen, Shanpu Yang, Yan Zhou, Jinɡjinɡ Li

**Affiliations:** ^1^ Department of Pediatrics, Yuhuan People’s Hospital, Taizhou, China; ^2^ Department of Laboratory Medicine, Yuhuan People’s Hospital, Taizhou, China

**Keywords:** allergic asthma, cytokine, children, macrophage, respiratory infection, inhaled corticosteroids (ICS), subcutaneous specific allergen immunotherapy (SCIT)

## Abstract

**Background:**

Exploring the characteristics of serum inflammatory cytokine changes during acute exacerbations of pediatric allergic asthma, and analyzing factors influencing poor asthma control and predictive indicators.

**Methods:**

Forty children with acute exacerbations of allergic asthma, either outpatients or inpatients, were selected as the observation group, and 40 healthy children undergoing physical examinations during the same period served as the control group. Flow cytometry was used to analyze the characteristics of blood inflammatory cytokines in both groups. Logistic multiple regression was used to analyze the influencing factors of poor asthma control, and ROC curve analysis was used to evaluate the indicators for predicting poor asthma control.

**Results:**

There were statistically significant differences in the levels of IL-2, IL-4, IL-10, IL-13,IFN-γ, TNF-α, as well as the ratios of IL-2/IL-4, IL-2/IL-5, IL-2/IL-10, IL-2/IL-13, IFN-γ/IL-4, IFN-γ/IL-5, IFN-γ/IL-13, TNF-α/IL-4, TNF-α/IL-5, TNF-α/IL-6, TNF-α/IL-13 in the peripheral serum, and the expression of CD86, CD206, and CD86/CD206 in peripheral blood mononuclear cells (PBMCs) between the observation group and the control group (*P* < 0.05). Univariate analysis indicated that respiratory infections, exposure to allergens, irregular use of inhaled corticosteroids (ICS), and peripheral blood eosinophil counts may be associated with poor asthma control (*P* < 0.05). Receiving Subcutaneous Specific Allergen Immunotherapy (SCIT) may serve as a protective factor against poor asthma control (*P* < 0.05). Logistic multiple regression analysis showed that respiratory infections and irregular use of ICS were independent risk factors for poor asthma control (*P* < 0.05), while SCIT was an independent protective factor against poor asthma control (*P* < 0.05). ROC curve analysis shows that IL-13 has a high accuracy in predicting poor asthma control, with areas under the curve of 0.741.

**Conclusions:**

In pediatric allergic asthma exacerbations, there is a decrease in the activity of Th1 cytokines and an increase in the activity of Th2 cytokines in the peripheral blood, accompanied by enhanced polarization of macrophages towards the M2 phenotype. Respiratory infections and irregular use of ICS are independent risk factors for poor asthma control, whereas SCIT is an independent protective factor against poor asthma control. IL-13 has high accuracy in predicting poor asthma control.

## 1 Introduction

Asthma is a heterogeneous disease characterized by chronic airway inflammation and airway hyperresponsiveness, presenting with diverse clinical phenotypes. Allergic asthma is a significant phenotype among them, accounting for up to 60%–80% of pediatric asthma cases (1–3). House dust mites are common inhalant allergens (4, 5). The incidence of asthma is on the rise globally, affecting approximately 358 million people (6). In China, the disease burden caused by asthma has been increasing over the past few decades (7, 8). Asthma not only affects the quality of life of affected children, but also has adverse effects on their mental health, academic performance, and social activities. In the treatment of asthma, achieving and maintaining control over asthma symptoms is the primary objective. Common treatment methods for pediatric asthma include medications, nebulization therapy, specific allergen immunotherapy, and others. However, due to the complexity of asthma's pathogenesis and the numerous triggering factors, coupled with poor medication adherence among some patients, many children with asthma are prone to acute exacerbations because of inadequate symptom control, thereby jeopardizing their lives (9–11).This study primarily investigates the characteristics of changes in serum inflammatory cytokines during acute exacerbations of allergic asthma in children, and analyzes the influencing factors and predictive indicators for poor asthma control.

## 2 Materials and methods

### 2.1 Patient characteristics

This study enrolled 40 children with acute exacerbations of allergic asthma as the observation group at Yuhuan People's Hospital from January 2023 to August 2024. These children included those seeking emergency care, outpatient services, or hospitalization. Diagnostic criteria for allergic asthma (12): (1) The presence of variable clinical symptoms such as wheeze, shortness of breath, chest tightness, cough, objective evidence of variable airflow limitation, and exclusion of other diseases that may cause asthma like symptoms; (2) Exposure to allergens can induce or worsen symptoms; (3) The allergen skin prick test or serum sIgE test should show a positive reaction for at least one allergen. The definition of acute exacerbation of asthma in this study refers to the acute exacerbation characterized by wheezing, coughing, chest tightness, and difficulty breathing, which requires additional medical interventions such as emergency visits, hospitalization, or outpatient treatment adjustment. Inclusion criteria: (1) Age 5–12 years old; (2) The allergic asthma that requires diagnosis by a pediatric respiratory specialist; (3) Parents are willing to be included in the research group with informed consent. Exclusion criteria: Individuals with bronchopulmonary dysplasia, congenital heart disease, pulmonary tuberculosis, bronchial foreign bodies, other congenital malformations, and immunodeficiency. Control group: 40 healthy children (without asthma and other diseases) aged 5–12 years who underwent physical examination in our hospital during the same period were selected. This study has been reviewed and approved by the Ethics Committee of Yuhuan People's Hospital (No: 2022-061).

### 2.2 Cytokine detection

All study subjects had 3 ml of venous blood drawn on an empty stomach and immediately sent to the laboratory for centrifugation at 3,600 r/min for 10 min. The separated serum was stored in a −80℃ freezer for testing. Flow cytometry was used to detect IL-2, IL-4, IL-5, IL-6, IL-10, IL-13, IFN-γ, and TNF-α (reagents produced by BioLegend).

### 2.3 CD86 and CD206 detection

PBMCs were isolated from the peripheral blood of asthmatic children and healthy control children using Ficoll density gradient centrifugation. All cells were stored in serum-free and animal protein free cell cryoculture medium (NCM Biotech) and kept at −80℃. Detect the expression of CD86 and CD206 (reagents produced by BioLegend) molecules using flow cytometry.

### 2.4 Allergen and blood eosinophil detection

The detection of specific IgE antibodies for inhalant and food allergens uses the EUROIMMUN Blot method, with test kits produced by EUROIMMUN Medizinische Labordiagnostika AG. The peripheral blood eosinophil count was detected by the XE-2100 fully automatic blood analyzer produced by Sysmex company.

### 2.5 Questionnaire survey

Using a s**e**lf-designed survey questionnaire, collect basic information, relevant lifestyle and dietary habits, and medical history characteristics of children. The basic information mainly includes age, gender, time of visit, and measurements of height and weight. It also includes detailed inquiry and record of respiratory tract infection and its pathogen during acute exacerbation of asthma, allergen test results (IgE-mediated allergy with positive inhalant or food allergen test results), exposure to allergens, regular use of ICS status, vitamin D level, season of onset, exposure to passive smoking, and living environment (whether it is close to the road or industrial area). Additionally, recent emotional state, whether there is SCIT treatment (In this study, SCIT with house dust mite (HDM) allergen preparations was only administered to children with asthma who were sensitized to dust mites. Children with food allergies, animal hair allergies, and other types of allergies were not included in the SCIT treatment group), recent C-ACT score for asthma control in children, and pulmonary ventilation function are also included.

### 2.6 Statistical analysis

The Shapiro–Wilk normality test is used to determine whether the data follows a normal distribution. The values are expressed as mean ± standard deviation or median and quartile range. Mann–Whitney *U* test is used for non normally distributed data. Normal distribution variables are tested using *t*-test. Single factor analysis was performed using the chi square test, while multiple factor analysis was performed using logistic regression analysis. A significance level of *P* < 0.05 was considered statistically significant. Construct Receiver Operating Characteristic (ROC) curves to evaluate and compare the discriminative performance of different variables.

## 3 Results

### 3.1 General information and characteristics of blood inflammatory cytokines in the observation group and control group

There were 40 cases in the observation group, including 19 males and 21 females, with an average age of 7.5 ± 1.8 years, a minimum age of 5 years, a maximum age of 12 years, an average weight of 27.3 ± 7.9 kg, a minimum weight of 18 kg, a maximum weight of 52 kg, an average height of 126.3 ± 12.1 cm, a minimum height of 107 cm, and a maximum height of 156 cm. Among the 40 asthma patients in the observation group, 24 cases showed positive results for inhalant allergens alone. Specifically, 19 cases were positive for dust mites, 2 cases were positive for both dust mites and cat hair, 1 case was positive for both cat hair and dog hair, 1 case was positive for fungi, and 1 case was positive for pollen from trees like poplar, willow, or elm. Additionally, there were 10 cases of pure food allergies. Among these, 5 cases were positive for allergies to both chicken egg and milk protein, 2 cases were positive for chicken egg allergy alone, 2 cases were positive for milk protein allergy alone, and 1 case was positive for allergies to both crab and shrimp. Furthermore, 6 cases showed positive results for both inhalant and food allergies. Specifically, 2 cases were allergic to dust mites combined with milk protein, 1 case was allergic to dust mites combined with chicken egg, 1 case exhibited allergies to cat hair, dog hair, chicken egg, and milk protein simultaneously, 1 case was allergic to fungi, chicken egg, crab, and shrimp, and 1 case was allergic to dust mites combined with shrimp. There were 40 cases in the control group, including 22 males and 18 females, with an average age of 8.0 ± 2.0 years, a minimum age of 5 years, a maximum age of 12 years, an average weight of 29.0 ± 7.2 kg, a minimum weight of 19 kg, a maximum weight of 51 kg, an average height of 128.5 ± 14.3 cm, a minimum height of 106 cm, and a maximum height of 162 cm. There was no statistically significant difference in general information such as gender, age, weight, and height between the observation group and the control group (*P* > 0.05). There were statistically significant differences in the levels of IL-2, IL-4, IL-10, IL-13,IFN-γ, TNF-α, as well as the ratios of IL-2/IL-4, IL-2/IL-5, IL-2/IL-10, IL-2/IL-13, IFN-γ/IL-4, IFN-γ/IL-5, IFN-γ/IL-13, TNF-α/IL-4, TNF-α/IL-5, TNF-α/IL-6, TNF-α/IL-13 in the peripheral serum, and the expression of CD86, CD206, and CD86/CD206 in peripheral blood mononuclear cells (PBMCs) between the observation group and the control group (*P* < 0.05), while there were no statistically significant differences in IL-5, IL-6, IL-2/IL-6, IFN-γ/IL-6, IFN-γ/IL-10 and TNF-α/IL-10 (*P* > 0.05) (Table 1).

**Table 1:** Characteristics of blood inflammatory factors in the observation group and control group.

Characteristic	Observation group (*n* = 40)	Control group (*n* = 40)	*P*
Sex (male)	19 (47.5)	22 (55.0)	0.655
Age (years)	7.5 ± 1.8	8.0 ± 2.0	0.322
Weight (kg)	27.3 ± 7.9	29.0 ± 7.2	0.324
Height (cm)	126.3 ± 12.1	128.5 ± 14.3	0.450
CD86	1,212.5 ± 169.7	1,308.5 ± 230.9	0.037
CD206	808.3 ± 126.3	707.4 ± 145.6	0.001
CD86/CD206	1.5 ± 0.3	1.9 ± 0.4	<0.001
IL-2 (pg/ml)	1.0 (0.8–1.2)	1.3 (1.1–1.8)	<0.001
IL-4 (pg/ml)	10.9 (4.0–52.0)	5.3 (3.8–14.2)	0.041
IL-5 (pg/ml)	2.3 (1.5–5.9)	2.1 (1.4–3.1)	0.146
IL-6 (pg/ml)	8.4 (5.7–14.3)	12.1 (5.8–20.6)	0.170
IL-10 (pg/ml)	1.8 (1.1–2.5)	4.8 (2.3–12.0)	<0.001
IL-13 (pg/ml)	11.2 (6.6–14.7)	3.6 (2.0–8.0)	<0.001
IFN-γ (pg/ml)	9.3 (8.0–13.7)	13.5 (8.3–27.2)	0.017
TNF-α (pg/ml)	7.2 (4.0–20.2)	12.6 (6.7–96.8)	0.001
IL-2/IL-4	0.1 (0.0–0.2)	0.2 (0.1–0.3)	0.003
IL-2/IL-5	0.4 (0.2–0.7)	0.6 (0.4–1.3)	0.002
IL-2/IL-6	0.1 (0.1–0.2)	0.1 (0.1–0.2)	0.333
IL-2/IL-10	0.5 (0.4–0.7)	0.3 (0.1–0.6)	0.028
IL-2/IL-13	0.1 (0.1–0.1)	0.4 (0.2–0.7)	<0.001
IFN-γ/IL-4	1.3 (0.2–2.4)	2.1 (1.0–4.6)	0.012
IFN-γ/IL-5	4.2 (1.5–6.7)	7.5 (4.1–11.2)	<0.001
IFN-γ/IL-6	1.2 (0.6–1.3)	1.0 (0.5–3.7)	0.504
IFN-γ/IL-10	5.6 (3.1–9.2)	3.0 (1.1–6.7)	0.054
IFN-γ/IL-13	0.8 (0.5–1.6)	3.4 (2.2–7.9)	<0.001
TNF-α/IL-4	0.9 (0.2–1.5)	2.1 (13–8.2)	<0.001
TNF-α/IL-5	3.0 (1.7–7.8)	7.1 (3.4–56.7)	<0.001
TNF-α/IL-6	1.0 (0.3–2.2)	2.0 (0.6–8.0)	0.021
TNF-α/IL-10	6.5 (2.4–8.8)	5.4 (2.9–10.5)	0.697
TNF-α/IL-13	0.7 (0.3–2.6)	4.0 (2.8–17.0)	<0.001

The data is expressed as mean ± standard deviation, median (interquartile range), and *n* (%); *t*-test is used for normally distributed variables, Mann-Whitney *U* test is used for non normally distributed data, and chi square test is used for categorical variables.

### 3.2 Univariate analysis of poor control of allergic asthma

Univariate analysis of the reasons for poor control of allergic asthma in children showed that respiratory infections, exposure to allergens, irregular use of ICS, and peripheral blood eosinophil counts may be associated with poor asthma control (*P* < 0.05). Receiving SCIT may be a protective factor against poor asthma control (*P* < 0.05). However, vitamin D levels, season, passive smoking, living environment, and emotional status were not found to be correlated with poor asthma control (*P* > 0.05) (Table 2).

**Table 2:** Univariate analysis of poor control of allergic asthma.

Factor	Variable value	*n*	Poorly controlled asthma	*P*
Respiratory tract infection	Yes	18	13 (72.2)	0.001
	No	22	4 (18.2)	
Allergen exposure	Yes	12	9 (75.0)	0.013
	No	28	8 (28.6)	
Irregular inhalation of ICS	Yes	15	12 (80.0)	<0.001
	No	25	5 (20.0)	
Vitamin D value	Yes	13	8 (61.5)	0.171
	No	27	9 (33.3)	
Spring and autumn season	Yes	21	12 (57.1)	0.062
	No	19	5 (26.3)	
Passive smoking	Yes	8	5 (62.5)	0.250
	No	32	12 (37.5)	
Eosinophilic counts	Yes	22	14 (63.6)	0.004
	No	18	3 (16.7)	
Live on the roadside or in an industrial area	Yes	9	5 (55.6)	0.456
	No	31	12 (38.7)	
Emotional instability	Yes	9	6 (66.7)	0.134
	No	31	11 (35.5)	
Receive specific immunotherapy	Yes	21	4 (19.0)	0.003
	No	19	13 (68.4)	

The chi-square test is used for categorical variables.

### 3.3 Multivariate analysis of poor control of allergic asthma

Multivariate logistic regression analysis conducted on the basis of univariate factors revealed that respiratory infections and irregular use of ICS are independent risk factors for poor asthma control (*P* < 0.05), while SCIT is an independent protective factor against poor asthma control (*P* < 0.05) (Table 3).

**Table 3:** Multivariate analysis of poor control of allergic asthma.

Variable	*β*	SE	Wald*χ*^2^	*P*	OR	95% CI
Respiratory tract infection	4.009	1.784	5.048	0.025	55.114	1.669–1,820.450
Allergen exposure	1.924	1.477	1.699	0.192	6.850	0.379–123.740
Irregular inhalation of ICS	4.274	1.676	6.507	0.011	71.818	2.692–1,916.231
Eosinophilic counts	1.225	1.537	0.636	0.425	3.405	0.167–69.227
Receive specific immunotherapy	−4.197	1.694	6.136	0.013	0.015	0.001–0.416

Multivariate analysis using binary regression analysis.

### 3.4 The predictive value of IL-13 for poor asthma control

**Figure 1:**
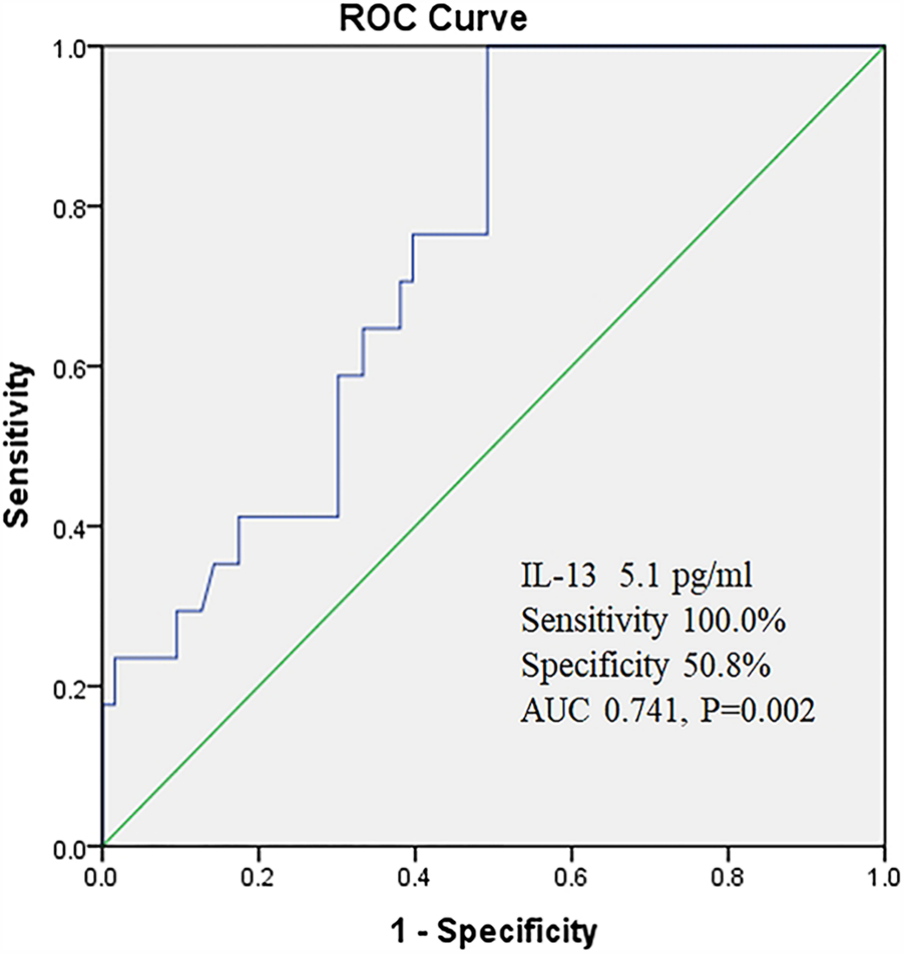
The predictive value of IL-13 for poor asthma control.

The ROC curve was used to evaluate the predictive value of IL-13 for poor asthma control. When the critical value of IL-13 was set at 5.1 pg/ml, the sensitivity was 100.0%, the specificity was 50.8%, and the area under the curve (AUC) was calculated to be 0.741 (95% CI: 0.626–0.856) (Figure1).

## 4 Discussion

Despite ongoing updates and dissemination of guidelines or consensus by the Global Initiative for Asthma (GINA) and various countries on the diagnosis, assessment, treatment, and management of pediatric asthma, the frequency of visits and hospitalization rates for acute asthma exacerbations in children has not declined (13, 14). Therefore, analyzing the risk factors and predicting the risks associated with poor asthma control in children is of great significance for preventing acute asthma exacerbations. In this study, by comparing the serum levels of inflammatory cytokines between children with allergic asthma exacerbations and healthy children (without asthma and other diseases), combined with Logistic multivariate regression analysis and ROC curve evaluation, we obtained a series of valuable findings. These findings not only enhance our understanding of the pathogenesis of allergic asthma in children but also provide new insights for clinical treatment and disease management.

Firstly, this study observed that the activity of peripheral blood Th1 cytokines (such as IL-2, IFN-γ) was significantly reduced, while the activity of Th2 cytokines (such as IL-4, IL-13) was significantly enhanced in children with acute exacerbations of allergic asthma. This result is consistent with previous research reports (15–17), further confirming the central role of Th1/Th2 immune imbalance in the pathogenesis of asthma. Overexpression of Th2 cytokines can promote airway inflammation, mucus secretion, and airway hyperresponsiveness, thereby exacerbating asthma symptoms. It is worth noting that this study also discovered some new inflammatory factor ratios (such as IL-2/IL-4, IL-2/IL-13, IFN-γ/IL-4, etc.) that showed significant differences between the two groups. These ratios may more sensitively reflect the degree of Th1/Th2 immune imbalance, providing a new perspective for the study of the pathogenesis of asthma.

In addition, this study also focused on the role of macrophages in the pathogenesis of asthma. Macrophages, as important immune cells, play a dual role in inflammatory responses. On the one hand, they can release pro-inflammatory cytokines, exacerbating the inflammatory response; On the other hand, they can also secrete anti-inflammatory factors to promote inflammation resolution and tissue repair (18, 19). This study found that in children with acute exacerbations of allergic asthma, there is an enhanced polarization of macrophages towards the M2 phenotype, characterized by decreased expression of CD86 and increased expression of CD206. M2 macrophages exhibit anti-inflammatory and pro-repair properties; however, their excessive polarization may lead to airway remodeling and disease chronicity. Therefore, balancing the polarization state of macrophages may represent a new direction for future asthma treatment.

When exploring the influencing factors of poor asthma control, this study found that respiratory infections and irregular use of ICS are two important independent risk factors. Respiratory infections are a frequent trigger for acute exacerbations of asthma, which can trigger or exacerbate airway inflammation and worsen asthma symptoms. As a first-line medication for asthma treatment, the irregular use of ICS may lead to poor disease control. This discovery emphasizes the importance of standardized treatment and management of respiratory infections in children with asthma, as well as the necessity of ensuring that children adhere to medical advice and use ICS regularly. Furthermore, of the 40 children with allergic asthma in this study, 60.0% were sensitized to dust mites. Specifically, among those sensitized to dust mites, 21 children—either solely sensitized or having dust mites as their primary allergen—received SCIT. Additionally, this study found that undergoing SCIT is an independent protective factor against poor asthma control. In recent years, with deepening understanding of asthma and allergic diseases, the role of specific immunotherapy in asthma treatment has gradually increased. A growing body of research evidence (20–22) has shown that specific immunotherapy not only alleviates asthma symptoms but also improves patients' quality of life and may potentially reduce the risk of acute exacerbations of asthma. Furthermore, specific immunotherapy holds the potential to prevent the onset of asthma and its further deterioration (23–25).

In terms of predictors for poor asthma control, this study found that IL-13 demonstrated high accuracy. ROC curve analysis revealed that IL-13 had an area under the curve (AUC) of 0.741, with a sensitivity of 100.0%. Although the specificity was only 50.8%, IL-13 still possesses certain clinical value in predicting poor asthma control. As an important member of the Th2 cytokine family, IL-13 plays a pivotal role in the pathogenesis of asthma (26, 27). It not only promotes airway inflammation and mucus secretion but also induces airway hyperresponsiveness and airway remodeling (28, 29). Therefore, monitoring IL-13 levels can assist clinicians in timely identifying children at high risk for poor asthma control and taking corresponding intervention measures. However, it is important to note that due to its relatively low specificity, IL-13 as a standalone predictor may have certain limitations. Future research can explore the combination of IL-13 with other inflammatory cytokines or biomarkers to enhance the accuracy of prediction. Additionally, univariate analysis in this study found that exposure to allergens and peripheral blood eosinophil count may be associated with poor asthma control, but they were not identified as independent risk factors. Factors such as vitamin D levels, season, passive smoking, living environment, and emotional status did not show significant associations with poor asthma control, which is inconsistent with previous research findings (30–34). This discrepancy may be related to factors such as sample size, study design, or regional differences. Future research can further expand the sample size, optimize study design, or conduct multicenter studies to more comprehensively assess the impact of these factors on asthma control.

In summary, this study offers a scientific rationale for deepening the understanding of asthma pathogenesis, optimizing treatment protocols, and enhancing disease control rates by comprehensively examining the characteristics of serum inflammatory cytokine changes during acute exacerbations of allergic asthma in children, as well as analyzing the pivotal factors and predictive indicators that influence asthma control. Future research endeavors can further explore the roles of additional inflammatory cytokines, macrophages, and genetic factors in asthma pathogenesis, alongside the efficacy and safety of novel therapeutic interventions, to provide more comprehensive guidance for the prevention and management of allergic asthma in children. Additionally, reinforcing health education for patients and their families to elevate their awareness and management capabilities concerning asthma represents a vital approach to improving asthma control outcomes.

## 5 Conclusions

During acute exacerbations of allergic asthma in children, there is a decrease in the activity of Th1 cytokines and an increase in the activity of Th2 cytokines in the peripheral blood, accompanied by enhanced polarization of macrophages towards the M2 phenotype. Respiratory infections and irregular use of ICS are independent risk factors for poor asthma control, whereas SCIT is an independent protective factor against poor asthma control. IL-13 has high accuracy in predicting poor asthma control.

## Data Availability

The original contributions presented in the study are included in the article/Supplementary Material, further inquiries can be directed to the corresponding author.
